# Tyrosine Phosphorylation Modulates the Vascular Responses of Mesenteric Arteries from Human Colorectal Tumors

**DOI:** 10.1155/2013/545983

**Published:** 2013-11-10

**Authors:** Eduardo Ferrero, María Dolores Mauricio, Miriam Granado, Oscar García-Villar, Martín Aldasoro, José María Vila, Manuel Hidalgo, Jorge Luis Ferrero, Nuria Fernández, Luis Monge, Ángel Luis García-Villalón

**Affiliations:** ^1^Departamento de Cirugía General y Digestiva (Seccion B), Hospital Universitario “12 de Octubre”, Universidad Complutense, Avenida de Córdoba, s/n, 28041 Madrid, Spain; ^2^Departamento de Fisiología, Universidad de Valencia, Avenida Blasco Ibáñez 15, 46010 Valencia, Spain; ^3^INCLIVA, Instituto Investigación Sanitaria, Hospital Clínico Universitario, Avenida Blasco Ibáñez 15, 46010 Valencia, Spain; ^4^Departamento de Fisiología, Facultad de Medicina, Universidad Autónoma de Madrid, Arzobispo Morcillo 2, 28029 Madrid, Spain

## Abstract

The aim of this study was to analyze whether tyrosine phosphorylation in tumoral arteries may modulate their vascular response. To do this, mesenteric arteries supplying blood flow to colorectal tumors or to normal intestine were obtained during surgery and prepared for isometric tension recording in an organ bath. Increasing tyrosine phosphorylation with the phosphatase inhibitor, sodium orthovanadate produced arterial contraction which was lower in tumoral than in control arteries, whereas it reduced the contraction to noradrenaline in tumoral but not in control arteries and reduced the relaxation to bradykinin in control but not in tumoral arteries. Protein expression of VEGF-A and of the VEGF receptor FLT1 was similar in control and tumoral arteries, but expression of the VEGF receptor KDR was increased in tumoral compared with control arteries. This suggests that tyrosine phosphorylation may produce inhibition of the contraction in tumoral mesenteric arteries, which may increase blood flow to the tumor when tyrosine phosphorylation is increased by stimulation of VEGF receptors.

## 1. Introduction

Tyrosine kinases are a family of protein kinases present in metazoans, which are mainly associated with receptors of growth factors such as vascular endothelial growth factor (VEGF), epidermal growth factor (EGF), and fibroblast growth factor (FGF) [1]. Tyrosine kinases have important roles in several cell functions as differentiation, proliferation, apoptosis, angiogenesis, and responses to neurotransmitters [2]. Tyrosine kinases also play a fundamental role in the development of malignant cells during cancer development, and therefore several inhibitors of tyrosine kinases have been added to the list of antitumoral agents available for cancer treatment [3].

Tyrosine phosphorylation may also regulate contraction of vascular smooth muscle. Vascular smooth muscle cells show levels of tyrosine kinase activity which are relatively high [4]. Inhibition of tyrosine phosphatase with sodium orthovanadate produced contraction of smooth muscles of rat aorta [5] or rat penile artery [6] and increased the contraction to hypoxia in sheep pulmonary veins [7] and to serotonin in rat basilar artery [8]. 

In tumoral tissues, tyrosine phosphorylation is often related to activation of growth factor receptors which have tyrosine kinase activity, including VEGF receptors which have a central role in cancer development [9]. VEGF acts on blood vessels mainly through two receptors: VEGF-R1/FLT1 and VEGF-R2/KDR, both of which have tyrosine kinase activity [10, 11]. Expression of VEFG is increased in tumors [12] and in the plasma of cancer patients [13, 14]. VEGF receptors also may be increased in tumors, either in the tumor cells or in the blood vessels supporting the tumor. Smith et al. [15] found that VEGFR-2 is overexpressed in blood vessels of colorectal, lung, and breast tumors, but not in the tumor cells. However, Amaya et al. [16] have reported increased KDR receptors in blood vessels and also in tumor cells of colorectal cancer. These studies have localized VEGF receptors in the microvasculature of the tumor and have not separated the expression according to the type of blood vessel, that is, artery, capillary, or vein. Most of the blood vessels in the microcirculation are capillaries and venules, which have little role in the regulation of blood flow, and there are not, to our knowledge, studies of the expression of VEGF or VEGF receptors specifically in the arteries supplying blood flow to the tumor.

Regulation of tumoral vasculature is an important factor in the development of solid tumors, as blood flow must increase in parallel with the growth of the tumor. The hypothesis of the present study is that tyrosine phosphorylation may play a role in the regulation of vasoconstriction and/or vasodilatation of the arteries supplying blood flow to the tumors. Therefore, we have studied the effect of increasing tyrosine phosphorylation on vasoconstriction and vasodilatation of arteries supplying colorectal tumors, comparing them with arteries supplying normal tissue. The inhibitors of tyrosine phosphatases such as vanadate increase tyrosine phosphorylation and reproduce the vascular effects of receptors with tyrosine kinase activity [17].

## 2. Methods

### 2.1. Collection of the Human Mesenteric Arteries

In this study, arteries (0.7–1.5 mm in external diameter) from 13 patients diagnosed with colorectal tumour were used (mean age: 72 ± 4 years, 7 males and 6 females). The study was approved by the local ethics committee and the informed consent from all the patients was obtained before they were allowed to participate. Arteries supplying blood flow to the tumour as well as arteries supplying the normal colon were dissected out at surgery from each patient and frozen on dry ice for RT-PCR and western blot techniques, or stored in cold isotonic saline solution for in vitro vascular response. 

### 2.2. Recording of Vascular Response

After collection and once transported to the laboratory, the arteries were cut into 2 mm long segments and each segment was prepared for isometric tension recording in a 4 mL organ bath containing modified Krebs-Henseleit solution at 37°C (mM): NaCl, 115; KCl, 4.6; KH_2_PO_4_, 1.2; MgSO_4_, 1.2; CaCl_2_, 2.5; NaHCO_3_, 25; glucose, 11. The solution was equilibrated with 95% oxygen and 5% carbon dioxide to a pH of 7.3-7.4. Briefly, two fine steel wires (100 *μ*m in diameter) were passed through the lumen of the vascular segment, one wire was fixed to the organ bath wall while the other was connected to a strain gauge for isometric tension recording (Universal Transducing Cell UC3 and Statham Microscale Accessory UL5, Statham Instruments, Inc.). This arrangement permits passive tension to be applied in a plane perpendicular to the long axis of the vascular cylinder. The changes in isometric force were recorded on a Macintosh computer using Chart v 3.6/s software and a MacLab/8e data acquisition system (ADInstruments). An optimal passive tension of 1 g was applied to the vascular segments, and then, they were allowed to equilibrate for 60–90 min. Before beginning the experiment, the vascular segments were stimulated with potassium chloride (50 mM) to determine the contractility of smooth muscle, and the segments which failed to contract at least 0.5 g were discarded.

To analyze the direct effect of tyrosine phosphorylation on the vascular tone of mesenteric arteries, cumulative dose-response curves were recorded for sodium orthovanadate (10^−4^–10^−2 ^M) in the vascular segments from arteries supplying the tumour (tumoral) and from those supplying normal colon (control) in the same patients. This effect was also recorded in the presence of the inhibitor of tyrosine kinase genistein (10^−4 ^M) to analyze whether the response was due to tyrosine phosphorylation. To study the effect of tyrosine phosphorylation in response to vasoconstrictor of vasodilator stimuli, the contraction to noradrenaline (10^−9^–10^−4 ^M) and to the endothelium-dependent vasodilator bradykinin (10^−9^–10^−5 ^M) was studied after treatment with orthovanadate (10^−3 ^M) in control and tumoral arteries. Orthovanadate was added to the organ bath 30 min before the concentration-response curve to noradrenaline or bradykinin, and before recording the relaxation to bradykinin, the arteries were precontracted with U46619 (10^−9^–10^−8 ^M) to reach a contractile tone of about 3–3.5 g.

Contraction in response to sodium orthovanadate and to noradrenaline was expressed as the percentage of the maximal contraction produced by potassium (50 mM), whereas the relaxation in response to bradykinin was expressed as the percentage of the active tone achieved with U46619. In each concentration-response curve, the pD_2_ was calculated as the negative logarithm of the concentration producing 50% of the maximal response by geometric interpolation.

### 2.3. RNA Isolation and RT-PCR Analysis

Total RNA was isolated from frozen vascular segment by using TriPure Isolation Reagent (Roche, Group SA) protocol, as suggested by the manufacturer. RNA concentration and integrity were determined using RNA 6000 Nano LabChip in an Agilent 2100 Bioanalyzer. The reverse transcription was performed with the High Capacity RNA-to-cDNA Master Mix (Applied Biosystems). The primers used for amplification of cDNA were obtained from TaqMan Gene Expression Assays (Applied Biosystems, inventoried assays) for VEGFA (Hs03929005_m1), VEGFR1 (FLT1) (Hs01052961_m1), VRGFR2 (KDR) (Hs00911700_m1), and GAPDH (endogenous control 4352934E). Amplification of target cDNA was done using a 7900HT Fast Real-Time PCR System with the following standardized thermal cycling conditions: 50°C for 2 min, 95°C for 10 min, followed by 40 cycles of 95°C for 15 sec and 60°C for 1 min. All RT-PCR reactions were run in duplicate. Threshold cycle (Ct) was determined for both target gene and GAPDH for each sample, and relative quantifying of both genes was determined with the 2-DDCt method [18].

### 2.4. Western Blot Analysis

The expression of VEGF-A, KDR, and FLT1 in vascular samples was measured by densitometry quantitation of immunoblots. Briefly, vascular tissues were homogenized in a lysis buffer (in mM): 50 Tris-HCl, 125 NaCl, 1 EDTA, 1 EGTA, 1% Nonidet (NP-40) containing 5% complete mini-tab cocktail protease inhibitor (Roche Biochemicals) and centrifuged at 10000 rpm for 15 min at 4°C. Protein concentration was determined using a modified Lowry method [19]. Then, 50 *μ*g of total protein was resolved in 12% SDS-PAGE and electrophoretically transferred onto a PVDF-membrane using Mini Trans-Blot cell (BioRad laboratories, CA, USA). Membranes were blocked in 5% skim milk for 1 h at room temperature. After an overnight incubation at 4°C using diluted monoclonal antibodies from Sigma (1 : 1000), the membranes were incubated with 1 : 2500 diluted horseradish peroxidase-conjugated secondary antibody (Sigma-Aldrich). The blots were then visualized using ImmunoStar HRP Substrate Kit (Bio-Rad) according to manufacturer's instructions. Relative densities of the bands were analyzed using Image Gauge v 4.0, Fujifilm. The proteins were normalized with tubulin. 

Antibodies are monoclonal anti-VEGF-A, monoclonal anti-VEGF receptor 1 (FLT1), monoclonal anti-VEGF receptor 2 (KDR), diluted at (1 : 1000), polyclonal anti-tubuline (1 : 1000) (Sigma Aldrich).

### 2.5. Statistical Analysis

The data are expressed as mean ± standard error of the mean. The responses to orthovanadate, noradrenaline, and bradykinin in tumour and control vascular segments, treated and untreated with genistein or orthovanadate, were compared by two-way ANOVA followed by Bonferroni test to determine which comparisons were statistically significant. Genic and protein expression of VEGF, FLT1, and KDR in control and tumoral arteries were compared by paired Student's *t*-test.

### 2.6. Compounds Used

The compounds used were (all from Sigma) sodium orthovanadate (Na_3_VO_4_); genistein (5,7-dihydroxy-3-(4-hydroxyphenyl)-4H-benzopyran-4-one, 4,5,7-trihydroxy-isoflavone); L-noradrenaline ((1R)-4-(2-amino-1-hydroxyethyl)-1,2-benzenediol); bradykinin acetate (Arg-Pro-Pro-Gly-Phe-Ser-Pro-Phe-Arg), U46619 (9,11-dideoxy-11*α*,9*α*-epoxymethano prostaglandin F2*α*).

## 3. Results and Discussion

**Figure 1:**
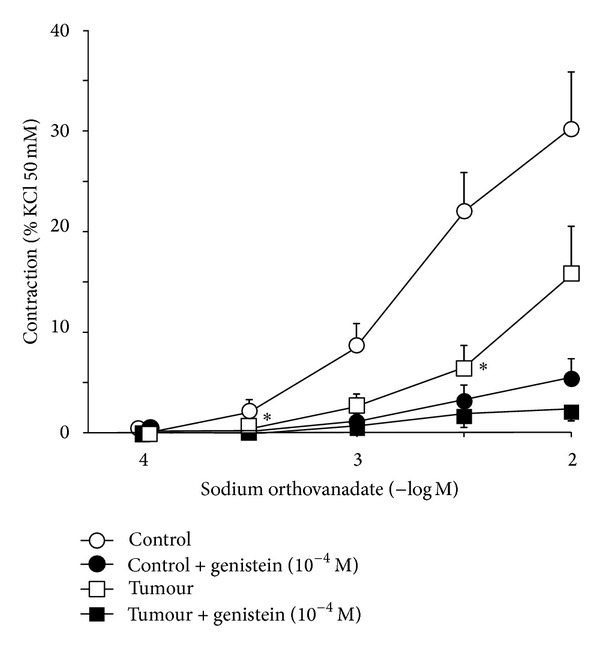
Contraction in response to sodium orthovanadate (10^−4^–10^−2 ^M) of mesenteric arteries supplying colorectal tumours (tumor, *n* = 10) and those supplying normal colon (control, *n* = 9), in the absence or in the presence of genistein (10^−4 ^M, *n* = 4). Values are represented as mean ± SEM. *P < 0.05 statistically significant versus control.

Stimulation with potassium chloride (50 mM) produced a similar contraction in tumoral (3.27 ± 0.22 g) and control (3.18 ± 0.28 g) arteries. Sodium orthovanadate produced concentration-dependent contraction, which was smaller in tumoral (*n* = 10) compared to control (*n* = 9) arteries (Figure 1). This contraction to orthovanadate was reduced by genistein, both in tumoral and control arteries (*n* = 4 in both groups).

**Figure 2:**
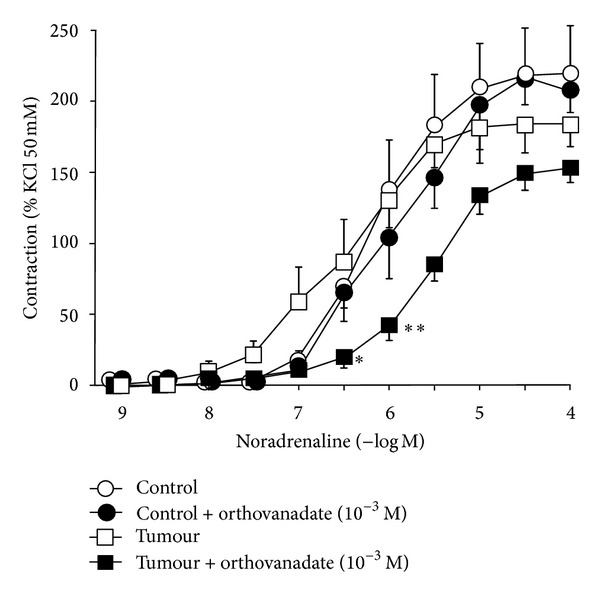
Contraction in response to noradrenaline (10^−9^–10^−4 ^M) of mesenteric arteries supplying colorectal tumours (tumour, *n* = 6) and those supplying normal colon (control, *n* = 6), untreated or treated (*n* = 6-7) with sodium orthovanadate (10^−3 ^M). Values are represented as mean ± SEM. **P < 0.01; *P < 0.05 statistically significant versus untreated segments.

Noradrenaline also produced concentration-dependent contraction, which was similar in control and tumoral arteries (pD_2_ = 6.20 ± 0.07, *n* = 6 versus 6.52 ± 0.23, *n* = 6, resp.) (Figure 2). In control arteries, pretreatment with sodium orthovanadate did not modify the contraction to noradrenaline (pD_2_ = 6.13 ± 0.35, *n* = 7), whereas in tumoral arteries, the contraction to noradrenaline in the segments treated with orthovanadate was smaller (pD_2_ = 5.68 ± 0.12, *n* = 6, *P* < 0.05) than that in untreated segments.

**Figure 3:**
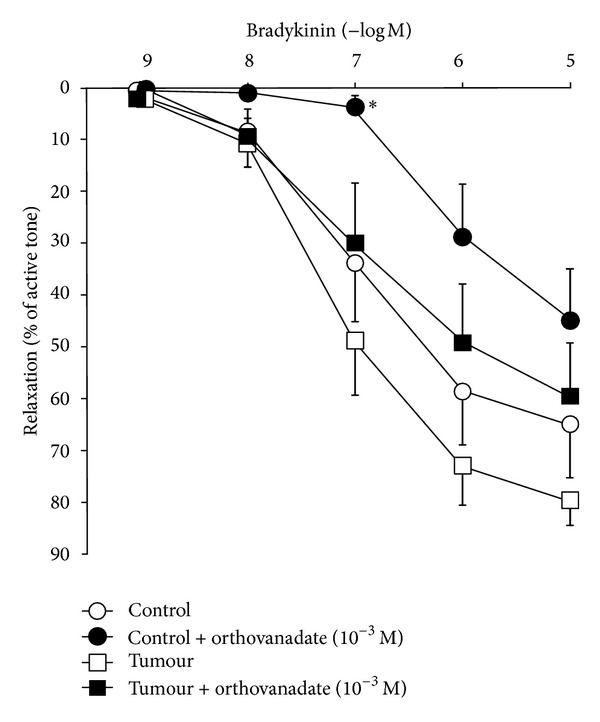
Relaxation in response to bradykinin (10^−9^–10^−5 ^M) of mesenteric arteries supplying colorectal tumours (tumour, *n* = 7) and those supplying normal colon (control, *n* = 7), precontracted with U46619 and untreated or treated (*n* = 8) with sodium orthovanadate (10^−3 ^M). Values are represented as mean ± SEM. *P < 0.05 statistically significant versus untreated segments.

In the segments precontracted with U46619, bradykinin produced concentration-dependent relaxation, which was similar in the control and tumoral arteries (pD_2_ = 6.89 ± 0.30, *n* = 7 versus 7.11 ± 0.24, *n* = 7, resp.) (Figure 3). Treatment with sodium orthovanadate reduced this relaxation in control arteries (pD_2_ = 6.00 ± 0.15, *n* = 8, *P* < 0.05); however, in tumoral arteries, this treatment did not modify the relaxation to bradykinin (pD_2_ = 6.67 ± 0.27, *n* = 8). The contractile tone produced by U46619 was similar in control and tumoral arteries, with and without pretreatment with sodium orthovanadate.

**Figure 4:**
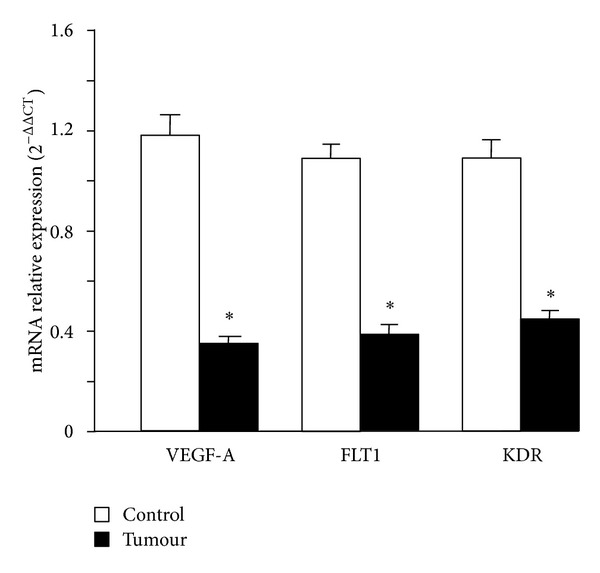
RT-PCR analysis of mRNA expression for VEGF-A, FLT1, and KDR in mesenteric arteries supplying colorectal tumours (tumour, *n* = 6) and those supplying normal colon (control, *n* = 6). Values were normalized to GAPDH mRNA expression. Data are presented as mean ± SEM. *P < 0.05 statistically significant versus control samples.

Quantitative PCR-RT analysis revealed a significant decrease in VEGF-A expression in tumor samples compared to the control (1.18 ± 0.20 versus 0.36 ± 0.06, *P* < 0.05). We have also determined the expression of KDR and FLT1, the main receptors of VEGF-A in the arteries [20]. Compared with the control, the vascular mRNA gene expression of KDR and FLT1 from tumor samples was significantly downregulated (60% and 64%, resp., *P* < 0.05). As a control, the expression of GAPDH was measured in parallel using the same mRNA samples (Figure 4).

**Figure 5:**
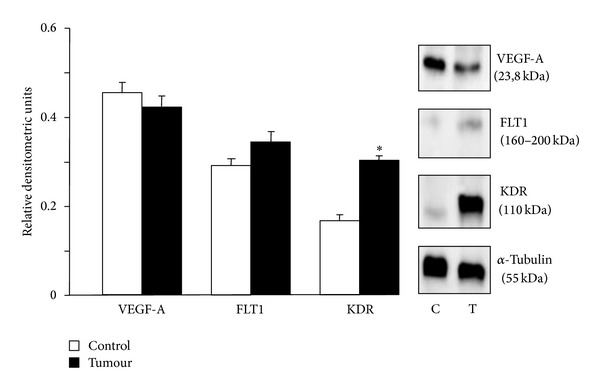
Protein expression in mesenteric arteries supplying colorectal tumours (T, *n* = 10) and those supplying normal colon (C, *n* = 10). VEGF-A, FLT1, KDR, and *α*-tubuline expression was determined by western blot. Data are presented as means ± SEM. *P < 0.05 statistically significant versus control samples.

Next we investigated the VEGF-A, FLT1, and KDR protein expression by western blot analysis. We found that protein expression of VEGF-A and FLT1 was similar in control and tumor vascular tissues. However, in samples from tumors, there is a significant increase in the protein expression of KDR (Figure 5).

This study finds that tyrosine phosphorylation may underlie a differential regulation of vascular function in colorectal tumors. Tyrosine phosphorylation is known to produce vasoconstriction of vascular [5] and nonvascular [21–24] smooth muscles. In our study, inhibition of tyrosine phosphatases by orthovanadate indeed produced vasoconstriction of mesenteric arteries. This contraction was inhibited by the tyrosine kinase inhibitor genistein, suggesting that it is due to increased tyrosine phosphorylation. The contraction produced by tyrosine phosphorylation has been related to Ca^2+^ influx [6, 25] or increased sensitivity to Ca^2+^ of the intracellular contractile mechanisms [26, 27].

Although orthovanadate contracted both control and tumoral arteries, the contraction was smaller in tumoral arteries, suggesting a reduced contractile effect of tyrosine phosphorylation in these arteries. Also, we have found that orthovanadate reduced the contraction to noradrenaline in tumoral arteries but not in control arteries. This contrasts with observations in rabbit aorta and mesenteric arteries, in which tyrosine phosphorylation increases adrenergic vasoconstriction [28]; therefore, the inhibitory effect observed in the present study may be specific for tumoral arteries. If tyrosine phosphorylation has an inhibitory effect in tumoral but not in normal arteries, it might also explain the small contraction produced by orthovanadate in tumoral arteries, as the inhibitory effect might counteract the vasoconstriction observed in control arteries. 

Orthovanadate also reduced endothelium-dependent vasodilatation in control mesenteric arteries. The effects of tyrosine phosphorylation on the endothelium may be mixed. Tyrosine kinases may enhance the release of endothelial nitric oxide [8, 29–33]. However, tyrosine phosphorylation has been also related to the endothelial dysfunction in diabetes [33], hyperhomocysteinemia [34], and Raynaud's phenomenon [35]. Our results agree with those latter studies, as in control mesenteric arteries, orthovanadate reduced endothelium-dependent vasodilatation to bradykinin. However, in tumoral arteries orthovanadate did not reduce significantly this relaxation. This may agree with a possible relaxing effect of tyrosine phosphorylation in these arteries, as we have hypothesized above, which could compensate for the relaxation impairment observed in control arteries. 

This possible inhibitory effect of tyrosine phosphorylation in tumoral arteries may be surprising, as the activation of tyrosine phosphorylation produces vasoconstriction in normal arteries [8, 36–39]. However, some receptors with tyrosine kinase activity may produce vasodilatation. Vascular endothelial growth factor (VEGF) produces vasodilatation in human [40], pig [41], or rat [42] coronary circulation, human placental circulation [43], bovine pulmonary arteries [44], and human mammary and radial arteries [45, 46]. Indeed, we have observed that application of VEGF produced relaxation of precontracted human mesenteric blood vessels (unpublished observations). Vanadate compounds are known to increase phosphorylation of VEGFR2/KDR receptors [47–49] and activate their effects [47, 49]. Therefore, as VEGFR2/KDR receptors may produce vasodilatation [50–52] and may be activated by vanadate, this may explain the inhibitory effect of orthovanadate observed in tumoral arteries. Also, we have found in this study that the expression of KDR receptors is increased in tumoral arteries and this might explain why this inhibitory effect is apparent in tumoral but not in control arteries. Expression of VEGFR-1/Flt1 [53, 54], VEGFR2/KDR [15, 55, 56], or both receptor subtypes [57–60] is increased in several types of tumors including colorectal ones [61–63]. However, the present study is the first, to our knowledge, showing increased expression of VEGF receptors in the arteries supplying blood flow to the tumor. These arteries are not mutated, while tumoral cells are, but may be altered by factors released in the microenvironment of the tumor [64]. It has been described that the arteries surrounding the tumor show differences in receptor expression compared with normal arteries from the same subject [65]. As it has been described that VEGF receptors are expressed mainly in the endothelium [66], this increased expression in tumoral arteries might explain why tyrosine phosphorylation has a partly inhibitory effect in these arteries but not in normal arteries. In normal arteries, tyrosine phosphorylation would produce contraction of smooth muscle, as we have observed, but in tumoral arteries, due to the increased expression of VEGF receptors, it would activate also relaxing mechanisms in endothelial cells. Contrasting with the fact that in tumoral arteries protein expression of FLT1 and KDR was unchanged or increased, respectively, the genic expression of these receptors was reduced in tumoral arteries. It has been shown that the VEGF may have opposite effects on protein and mRNA expression of FLT1 and KDR receptors in cultured human umbilical vein endothelial cells, which may be an adaptive mechanism to compensate for receptor desensitization and allow recovery of responsiveness to VEGF [64].

## 4. Conclusions

These results may have relevance, as the medium in the vicinity of the tumour may be rich in factors that activate tyrosine kinases and inhibitors of tyrosine kinase are used as antitumoral agents. Our results suggest that in normal arteries, tyrosine phosphorylation may produce vasoconstriction, whereas in the arteries supplying the tumor, it may produce inhibition of contraction. This may redistribute blood flow to the tumor, thus facilitating its growth.
